# Effects of salinity on gonadal development, osmoregulation and metabolism of adult male Chinese mitten crab, *Eriocheir sinensis*

**DOI:** 10.1371/journal.pone.0179036

**Published:** 2017-06-19

**Authors:** Xiaowen Long, Xugan Wu, Lei Zhao, Haihui Ye, Yongxu Cheng, Chaoshu Zeng

**Affiliations:** 1 College of Ocean and Earth Sciences, Xiamen University, Xiamen, China; 2 Centre for Research on Environmental Ecology and Fish Nutrition of Ministry of Agriculture, Shanghai Ocean University, Shanghai, China; 3 Shanghai Engineering Research Center of Aquaculture, Shanghai Ocean University, Shanghai, China; 4 National Demonstration Centre for Experimental Fisheries Science Education, Shanghai Ocean University, Shanghai, China; 5 College of Science & Engineering, James Cook University, Townsville, Queensland, Australia; Zhejiang University College of Life Sciences, CHINA

## Abstract

As a catadromous species, salinity is a key parameter that affects gonadal development of Chinese mitten crab *Eriocheir sinensis* during reproductive migration. It is however unclear the effects of salinity on the gonadal development of male *E*. *sinensis* as well as their physiological responses to salinity during reproductive migration. This study investigated the effects of four salinities (0 ‰, 6 ‰, 12 ‰ and 18 ‰) on gonadal development, osmoregulation and metabolism of adult male *E*. *sinensis* over a 40-day period. The results showed that elevating salinity promote gonadal development, increase hemolymph osmolality and K^+^ and Mg^2+^ concentrations (*P* < 0.05). The 12 ‰ salinity resulted in the highest contents of taurine and arginine in the hemolymph while the highest contents of threonine, phenylalanine, lysine, ß-alanine, tryptophan, ornithine and total free amino acids were found for 0 ‰ treatment (*P* < 0.05). A decreasing trend was detected for the Na^+^/K^+^-ATPase activity and its mRNA expression level in the posterior gills with salinity (*P* < 0.05). Total saturated fatty acids in the anterior gills decreased with increasing salinity (*P* < 0.05); the 0 ‰ treatment had the highest total polyunsaturated fatty acids in the posterior gills while total n-6 polyunsaturated fatty acids increased with salinity (*P* < 0.05). The hemolymph glucose and uric acid showed a decreasing trend as salinity while an increasing trend was found for the hemolymph triglyceride and high-density lipoprotein cholesterol (*P* < 0.05). The 12 ‰ treatment had the highest levels of hemolymph malonaldehyde and hepatopancreatic γ-glutamyltranspeptidase (*P* < 0.05). In conclusion, these results suggested that the brackish water promote gonadal development of male *E*. *sinensis*, and increase osmolality and ionic concentrations in hemolymph while reduced the activity of Na^+^ /K^+^- ATPase and its mRNA expression in the posterior gills as well as metabolism.

## Introduction

The Chinese mitten crab, *Eriocheir sinensis*, is a catadromous crustacean species, which distribute widely across East Asia from China to the Korean Peninsula and has a very complex life history: the juvenile *E*. *sinensis* spend most of their lives in freshwater, but after puberty molt, adult crabs must migrate downstream to estuaries for reproduction [1–3]. During the reproductive migration, the gonads of the crabs gradually develop and become mature in the estuaries where they copulate and subsequently spawn [3–5]. The females carry their eggs in the abdomen during incubation and hatching out larvae in estuaries, the newly hatched zoeal larvae develop through 5 instars to become megalopae who migrate upstream towards freshwater rivers and lakes [5, 6]. It hence can be inferred that salinity plays an important role on gonadal development and reproduction of *E*. *sinensis* [2, 7].

*E*. *sinensis* is known as a euryhaline species and a strong osmoregulator as they can function equally well in freshwater or brackish environments [8–9]. This can be attributed to its well-developed osmoregulatory capacity [10–11]. A number of studies have investigated the effects of short-term salinity acclimation or abrupt salinity changes on osmotic and ionic regulation, metabolism and immune responses of *E*. *sinensis* [10, 12–13]. However, very limited information is available on effects of longer term exposure to elevated salinity on adult *E*. *sinensis*. A previous study has shown that brackish water (15 ‰) accelerated gonadal development of *E*. *sinensis* [14]. Although a previous study has investigated the effects of long term salinity acclimation on osmoregulation and physiological metabolism of adult male *E*. *sinensi* [15], it is however unclear the effects of different salinities on gonadal development as well as physiological responses to salinity during gonadal development.

Previous studies have demonstrated that various inorganic ions, particularly sodium and chloride, are the main contributors to hemolymph osmolality of crustaceans [9, 16]. Meanwhile, free amino acids (FAAs) also contribute to hemolymph osmolality although their concentrations are generally lower than inorganic ions. Several specific FAAs (e.g. glycine, proline and alanine) are known as the main osmotic modulators as they play an important role in osmoregulation during salinity acclimation [17–18]. On the other hand, the Na^+^/K^+^-ATPase in the posterior gills is known as one of the most important enzymes for crustacean osmoregulation [9, 19]. Past studies have shown that gill fatty acid composition of crabs changed significantly during salinity acclimation, and such change may affect enzymatic activity and permeability of the gills, for instance, the decrease in polyunsaturated fatty acids (PUFAs) in the gills could reduce enzyme efficiency and permeability of the gills, hence likely less energy expenditures on osmoregulation [20–22].

Moreover, salinity affects other physiological processes of crustaceans, and there are various indices for evaluating their physiological responses and metabolism, which include glucose (GLU), superoxide dismutase (SOD) and acid phosphatase (ACP) [10, 12]. Although our previous study has preliminarily investigated the effects of long term salinity adaptation on the osmoregulation and physiological metabolism of adult male *E*. *sinensi* [15], however it remains unknown the effects of different salinities on hemolymph FAAs, gill Na^+^/K^+^-ATPase gene expression and fatty acid profile of *E*. *sinensis* during gonadal development. Hence, to date, there is a lack of comprehensive study on osmoregulation and physiological responses of *E*. *sinensis* to different salinity conditions.

It is well known that salinity in esturies fluctuates substantially, affected by tides and freshwater runoff from the rivers [13]. For example, salinity of Yangtze estuary reportedly ranges from 3.4 ‰ to 17 ‰ [5, 23]. Therefore as adult *E*. *sinensis* migrate towards estuaries, they are expected to activate osmoregulatory mechanisms to cope with the instable salinity environment [10, 13]. Previous studies have shown there are clear gender differences in the onset time of puberty molting, reproductive migration, and arriving time in the estuaries as well as estuarine salinity conditions when and where the crabs arrive between the male and the female *E*. *sinensis* [4, 23–24]. Such differences suggest that there could be gender differences in physiological responses to salinity change; hence the effects of salinity on males and female crabs should be studied separately. This study was hence conducted to investigate the effects of long-term exposure of different salinity on gonadal development, the hemolymph osmolality, major ions and free amino acids (FAAs) concentrations, gill Na^+^/K^+^-ATPase activity and its mRNA expression and fatty acid profile, and other physiological indices of adult male *E*. *sinensis*. It is expected that the results will shed new lights on adaptive mechanisms to salinity changes during reproductive migration of *E*. *sinensis*.

## Materials and methods

### Experimental design and setup

The experiment was conducted at Fengxian hatchery, research center of Shanghai Fisher Research Institute, Shanghai, China. During early October 2013, post puberty molting male *E*. *sinensis* were purchased from an enclosure crab farm in Yangchenghu Lake, Jiangsu province, China. Only healthy, active and intact crabs with immature gonads (initial mean gonadosomatic index (GSI) = 1.62%) were selected and randomly stocked into experimental tanks. The criteria used for determining mature vs. immature males are based mainly on following two features, which only found in matured males: 1) more than 80% area of the chelipeds is covered with hairs; and 2) male petasma has hardened. The initial body weight (BW) of the male crabs ranged between 150 to 160 g. The trial was conducted in 8 indoor polyethylene tanks (length × width × depth = 2.5 m × 3.75 m × 1.0 m). Approximately 40% bottom area of each tank was covered with 10–20 cm fine sand and polyvinyl chloride (PVC) tubes (diameter = 15 cm) and tiles were provided as shelters for the crabs. Four salinity treatments, i.e. 0 ‰, 6 ‰, 12 ‰ and 18 ‰, were set up and each treatment had two replicate tanks with 30 crabs per tank. During the experiment, water level in each tank was maintained at 70 cm. Prior to the experiment, the salinity in all tanks was 0 ‰, the salinity in designated tanks was subsequently increased to the designated levels by adding concentrated brine at a rate of 3 ‰ day ^-1^. This experiment started on 11^th^ October, 2013 and lasted 40 days.

During the experiment, all tanks were provided with continuous aeration. Photoperiod was set at 12 h light: 12 h dark with fluorescent lamps (40W) as the light source. The crabs were fed daily at 18: 00 with trash fish and the food residue was removed next morning. The amount of feeding was adjusted according to the water temperature and food residues: i.e. 3–5% of total biomass when water temperature > 20°C but reduced to 1–3% of total biomass when temperature was 15–20°C. The ammonia-N, nitrite, dissolved oxygen (DO) and pH were measured every three days and water was exchanged based on the measurements to maintain the water quality throughout the experiment as follows: ammonia-N < 0.5 mg L^-1^; nitrite < 0.15 mg L^-1^; DO level > 4 mg L^-1^ and pH 7.0–8.5.

### Sample collection

All crabs were fasted for 24 h from the end of day 40 until day 41 prior to sampling. Four crabs were randomly sampled from each tank and their weights were measured using a digital balance (precision = 0.01 g). Subsequently the crabs were treated with ice cold shock, and around 2 mL hemolymph was then withdrawn by inserting a 1.0 mL syringe at the base of the third walking limb of the crabs and stored in 2 mL tubes at—40°C for later analysis of osmolality, ions concentrations, free amino acids (FAAs) and other biochemical parameters. *E*. *sinensis* has six pairs of gills; previous studies have shown that the first 3 pairs of the gills (pairs 1–3, also known as anterior gills) are responsible for respiration while pairs 4–6 of posterior gills are specified for osmoregulation [25]. In this study, all 3 pairs of anterior and posterior gills were sampled and stored at -40°C for fatty acids analysis. For the Na^+^/K^+^-ATPase activity and its mRNA expression analysis in posterior gills, the pairs 5 were sampled and snap frozen in liquid nitrogen, and then stored at -80°C for late analysis. The gonad and hepatopancreas of each crab was also dissected out and weighted for calculating gonadosomatic index (GSI) and hepatosomatic index (HSI) before stored at -40°C for later analysis of biochemical parameters. The GSI and HSI were calculated using the following formulas:

$$GSI=\frac{M_{G}}{M_{C}}\times 100\%$$

$$HSI=\frac{M_{H}}{M_{C}}\times 100\%$$

where *M*_*G*_ is gonad weight, *M*_*H*_ is hepatopancreas weight and *M*_*C*_ is crab weight.

### Measurement of hemolymph osmolality and ionic concentrations

The hemolymph samples were thawed on ice, and the clot was disrupted using an IKA homogenizer (T10B, IKA Co., Germany) according to Henry et al. (2003) [26]. The hemolymph clot was then centrifuged at 10, 000×g for 20 min at 4°C, and the supernatant was collected for later analysis. The hemolymph osmolality was analyzed using a freezing-point osmometer (Osmomat 030, Gonotec, Berlin, Germany). The concentrations of Na^+^, K^+^, Ca^2+^ and Cl^-^ were determined by an electrolyte analyzer (K-Lite5, Meizhou Kangli high-tech Co., Ltd., Guangzhou, China), while the concentration of Mg^2+^ was determined with a spectrophotometer (T6 New Century, Beijing Purkinje General Instrument Co., Ltd, Beijing, China) at 540 nm absorbance, and the assays was performed using commercial assay kits (Nanjing Jiancheng Bioengineering Institute, Jiangsu, China) according to manufacture instructions.

### Free amino acid analysis

Each hemolymph sample was deproteinized by adding an equal volume of 12% (m: v) trichloroacetic acid (TCA) according to the method described by Chew et al. (1999) [27], and subsequently vibrated using SCILOGEX MX-S vortex mixer (SCILOGEX, LLC, Berlin, CT, USA) before being placed in 4°C freezer for 20 min. The mixture was then centrifuged at 10, 000×g and 4°C for 10 min with a centrifuge (Eppendorf 5417R, Eppendorf Co., Hamburg, Germany). The supernatant was collected and pH adjusted to 2.2 by adding 6 M NaOH solution. The analysis of FAAs was performed using a Hitachi L-8900 amino acid analyzer (Hitachi, Ltd., Tokyo, Japan) with a Li column (Inter diameter × length = 4.6 mm × 60 mm), which packed with Hitachi custom ion exchange resin #2622 (Particle size: 3μm).

### Na^+^/K^+^-ATPase activity and its mRNA expression

Approximately 0.2 g of posterior gills was homogenized in 1 mL of ice-cold homogenization buffer [1mol L^-1^ Tris-HCl (pH = 7.6): 10 mL; NaCl: 2.925 g; EDTA: 0.05 g; 100 mmol L^-1^ PMSF: 0.5 mL; and dilute to 500 mL with distilled water] by an IKA homogenizer (T10B, IKA, Co., Germany). The homogenate was centrifuged at 10, 000×g for 10 min at 4°C, and the supernatant was collected for the later analysis. The activity of Na^+^/K^+^-ATPase and total soluble protein in the supernatant were assayed by a spectrophotometer (T6 New Century, Beijing Purkinje General Instrument Co., Ltd, Beijing, China) and commercial assay kits (Nanjing Jiancheng Bioengineering Institute, Nanjing, China). A unit of ATPase activity was obtained as 1 μmol inorganic phosphate (P_i_) produced from the reaction in which ATP was resolved by ATPase in per mg tissue protein per hour (μ mol P_i_^-1^ hr ^-1^ mg protein ^-1^).

The frozen posterior gills were ground in a mortar with liquid nitrogen, and then total RNA was isolated using a RNA extracting kit (Cat. D9108A, Takara Biotechology Co., Ltd., Dalian, China) following the manufacture’s protocol. The final total RNA was dissolved in 200 mL RNase-free water. The concentration of total RNA was determined using a Nano-Drop 2000 spectrophotometer (Thermo, Scientific, USA), and the RNA integrity was checked with 1% agarose gel electrophoresis. One hundred ng of total RNA was used as reverse-transcription template for the synthesis of first strand cDNA using a reverse transcription kit (Cat.D2639A, Takara Biotechology Co., Ltd., Dalian, China). According to Na^+^/K^+^-ATPase gene sequence of *E*. *sinensis*, specific primers were designed by Primer Premier 5.0 software, and the sequence was shown in Table 1. The β-actin gene of *E*. *sinensis* was used as a reference gene.

**Table 1:** Primers for quantitative real-time PCR of *Na*^*+*^*/K*^*+*^*-ATPase α1* gene of adult male *E*. *sinensis*.

Primer	Sequence (5’- 3’)	NCBI accession number or reference
NAK-RT F	TGAATGACTCCCCAGCTCTCAAGA	AF3011581.1
NAK-RT R	CAGAATCATGTCAGCAGCCTGCTT	
*β-acting* 1 F	TCATCACCATCGGCAATGA	Guo et al.(2013) [28]
*β-acting* 1 R	TTGTAAGTGGTCTCGTGGATG	HM053699.1

### Fatty acid profile analysis

Prior to the fatty acid analysis, the anterior and poster gills of crabs from each experimental tank were pooled, homogenized and freeze-dried, respectively. Total lipids were extracted with chloroform-methanol (2:1, v/v) according to the method described by Folch et al. (1975) [29], which were subsequently esterified with boiling 14% boron trifluoride/methanol (w/w) [30].

Fatty acid methyl esters (FAMEs) were extracted by hexane. FAMEs were analyzed by flame ionization detection (FID) after injecting a sample into a Thermo Trace GC Ultra gas chromatograph fitted with a 100m × 0.25mm ID (0.2 *μm* film thickness) Supelco SP- 2560 capillary column (Supelco, Inc., Billefonte, PA, USA). Briefly, the injector and detector temperatures were kept at 260°C,while the column temperature was initially held at 70°C. It was then increased, at 50°C min ^-1^, to 140°C and held for 1min, followed by an increase at 4°C min ^-1^ to 180°C and held for 1min. It was then further increased at 3°C min ^-1^ to the final temperature of 225°C and held for 30 min until all FAMEs had been eluted. The total time used was 58.4 min. The carrier gas was nitrogen with the flow velocity at 1mL min ^-1^. The peaks were identified by comparing retention times with known standard (Sigma-Aldrich Co., St. Louis, MO, USA). Fatty acid profile was expressed as percentage of each fatty acid to the total fatty acids (% total fatty acids) based on the area percentage.

### Other biochemical parameters analysis

The hepatopancreas tissue plus the 5-fold volume (v/w) of icy physiological saline solution [210 mmol L^-1^ NaCl; 13.6 mmol L^-1^ KCl; 3.8 mmol L^-1^ MgCl_2_; 2.6 mmol L^-1^ Na_2_SO_4_; 10 mmol L^-1^ Hepse (pH 7.5)] were added to a 5 mL tube and homogenized using an IKA homogenizer (T10B, IKA, Co., Germany). The homogenate of each sample was centrifuged at 10, 000×g for 10 min at 4°C, and the supernatant was collected for the later analysis.

The total cholesterol (TC), triglyceride (TG), uric acid (UA), urea and glucose (GLU) were determined according to Qiu et al. (2011) [31] while the levels of high-density lipoprotein cholesterol (HDL-C) and low-density lipoprotein cholesterol (LDL-C) were measured according to Deng et al. (2011) [32]. The activities of alkaline phosphatase (ALP), γ-glutamyltranspeptidase (γ-GT), superoxide dismutase (SOD) and acid phosphatase (ACP) were analyzed with a spectrophotometer (T6 New Century, Beijing Purkinje General Instrument Co., Ltd, Beijing, China) at 37°C and 510, 405, 560, and 510 nm absorbance, respectively; and their assays were performed with detection kits (Suzhou Comin Biotechnology Co., Ltd., Jiangsu, China) according to manufacture instructions. The contents of malonaldehyde (MDA) in hemolymph and hepatopancreas were determined by the thiobarbituric acid method [33]. Total lipids in the hepatopancreas were extracted with chloroform-methanol (2:1, v/v) according to the method by Folch et al. (1957) [29].

### Statistical analysis

Data are presented as mean ± standard error (SE). All data was analyzed using one-way ANOVA after confirmation of normality and homogeneity of variance. If any significant differences were detected (*P* < 0.05), differences among treatments were identified using Duncan’s multiple range tests. For all statistical analysis the SPSS statistical package version 16.0 was used and significant relationship were determined when *P* < 0.05.

## Results

### Survivals, GSI and HSI

**Fig 1:**
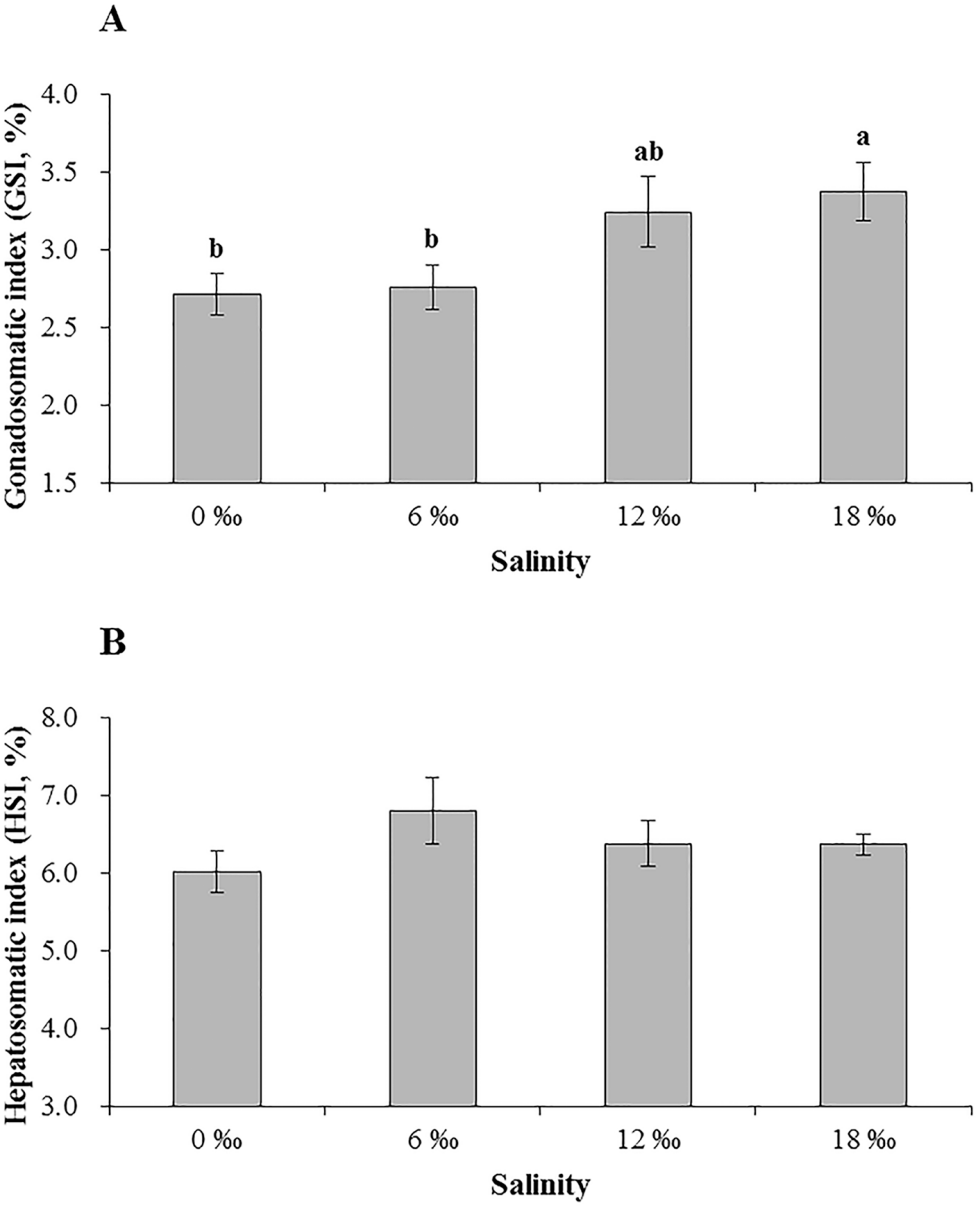
**The mean gonadosomatic index (GSI, %) (A) and hepatosomatic index (HSI, %) (B) of adult male *E*. *sinensis* subjected to different salinities**. Different letters on the tops of the bars indicated significant differences (*P* < 0.05).

The survival rates of the experimental crabs of the four salinity treatments were similar, ranged from 72% of the 18 ‰ treatment to 80% of the 0 ‰ treatment, no significant difference was found among all treatments (*P* > 0.05). An increasing trend was detected for the gonadosomatic index (GSI) of adult male *E*. *sinensis* with increasing salinity (Fig 1A). The crabs from 18 ‰ treatment had the significant higher GSI than the 0 ‰ and 6 ‰ treatments (*P* < 0.05) while no significant difference was found between 12 ‰ and 18 ‰ treatments (*P* > 0.05). On the other hand, salinity appeared to have no significant effect on hepatosomatic index (HSI) of the crabs (Fig 1B).

### Hemolymph osmolality, ionic concentrations and free amino acid composition

**Fig 2:**
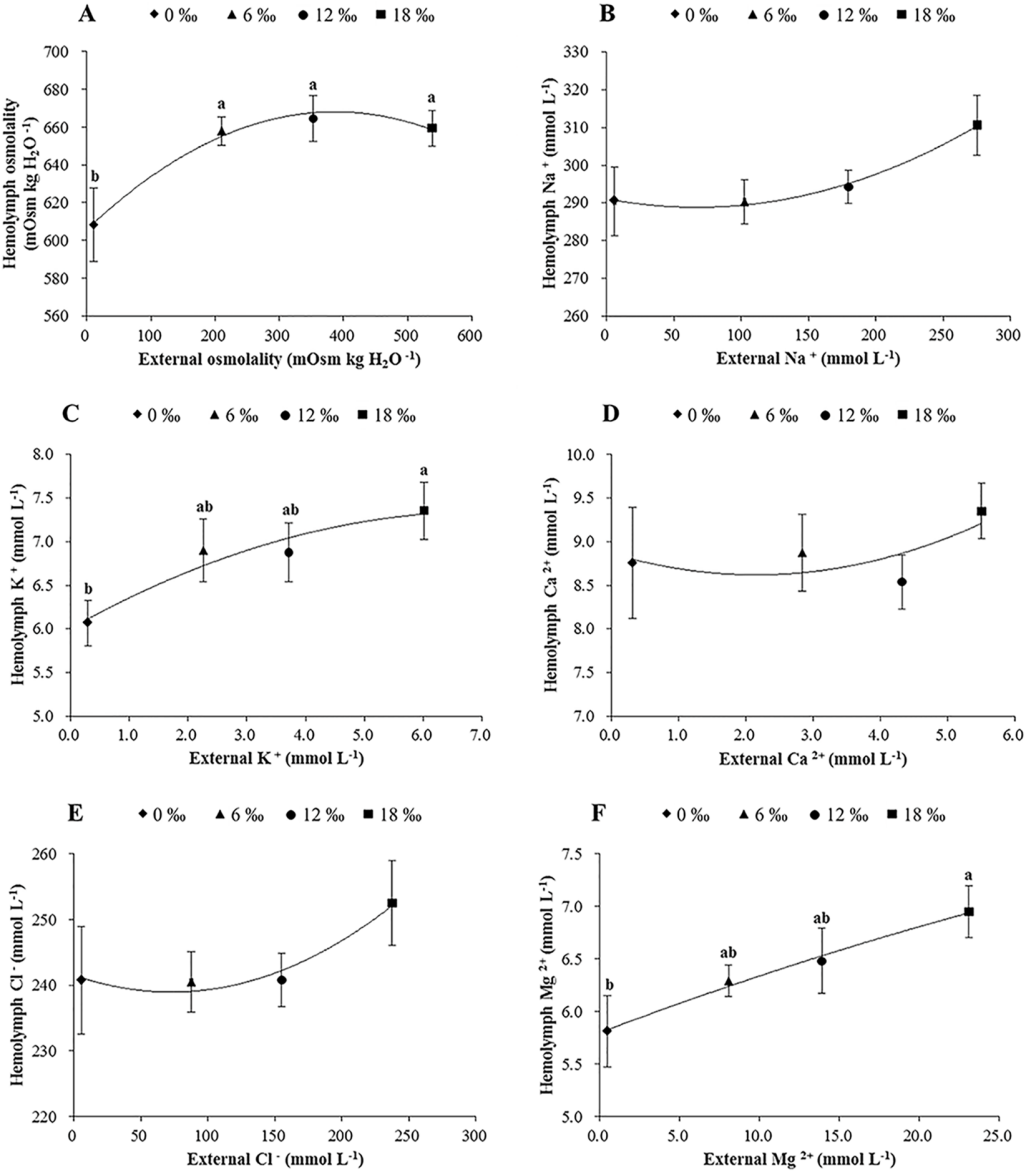
**The mean hemolymph osmolality (mOsm kg H**_**2**_**O** ^**-1**^**) (± SE) (A), Na**^**+**^ **(B), K**^**+**^ **(C), Ca**^**2+**^ **(D), Cl**^**-**^ **(E) and Mg**^**2+**^ **(F) (m mol L** ^**-1**^**) of adult male *E*. *sinensis* subjected to different salinities**. Different letters indicated significant differences (*P* < 0.05).

A trend of hemolymph osmolality first increased with the increasing salinity from 0 ‰ and 12 ‰ but then decreased slightly as salinity further increased to 18 ‰ was shown, resulting in the highest and the lowest osmolality detected for the 0 ‰ and the 12 ‰ treatments, respectively (*P* < 0.05, Fig 2A). As expected, all ion concentrations measured showed an overall increasing trend with increasing salinity, however no significant difference was detected among treatments for Na^+^, Cl^-^ and Ca^2+^ (*P* > 0.05, Fig 2B, 2D and 2E) while the concentrations of K^+^ and Mg^2+^ increased significantly with increasing salinity (*P* < 0.05, Fig 2C and 2F).

The crabs from salinity 12 ‰ treatment detected the highest levels of taurine, α-aminoadipicacid (α-AAA) and arginine in hemolymph (*P <* 0.05, Table 2). On the other hand, the highest contents of threonine, proline, alpha-amino-n-butyric acid (α-ABA), cystine, phenylalanine, lysine, cystathionine, ß-alanine, tryptophan, ornithine were found for the 0 ‰ treatment (*P* < 0.05). No significant differences were found for the contents of aspartic acid, β-aminoisobutyric acid and citrulline among all treatments (*P* > 0.05). Total free amino acids (TFAAs) showed a clear trend of decrease with increasing salinity, and the significant higher TFAAs was found for 0 ‰ treatment compared to the other treatments (*P* < 0.05).

**Table 2:** Free amino acids composition in hemolymph of adult male *E*. *sinensis* (nmol mL ^-1^) subjected to different salinities.

Free amino acids	Salinity			
	0 ‰	6 ‰	12 ‰	18 ‰
Taurine	375.00 ± 30.85^b^	441.67 ± 40.50^a^^b^	499.50 ± 26.50^a^	370.33 ± 30.60^b^
Aspartic acid	18.75 ± 3.35	19.67 ± 2.96	18.00 ± 2.65	21.67 ± 1.45
Threonine	86.50 ± 7.50^a^	63.00 ± 10.02^a^^b^	54.33 ± 10.67^b^	44.00 ± 3.06^b^
Serine	67.20 ± 12.98	54.50 ± 2.50	63.67 ± 7.17	55.33 ± 0.33
Asparagine	52.35 ± 13.29	57.10 ± 9.37	48.02 ± 5.52	42.31 ± 5.26
Glutamic acid	50.00 ± 7.29	59.67 ± 9.33	51.00 ± 6.11	50.00 ± 3.61
Glutamine	231.49 ± 61.33	205.54 ± 47.03	224.23 ± 44.38	186.76 ± 19.65
α-AAA	18.63 ± 2.25^b^	16.15 ± 1.65^b^	39.50 ± 2.10^a^	11.05 ± 3.31^b^
Proline	1146.00 ± 166.15^a^	860.63 ± 70.00^a^^b^	833.75 ± 101.65^a^^b^	690.29 ± 88.85^b^
Glycine	359.00 ± 48.59	313.33 ± 23.98	325.67 ± 58.60	265.67 ± 17.42
Alanine	613.00 ± 110.96	453.67 ± 38.56	412.00 ± 60.01	351.33 ± 39.94
Citrulline	8.10 ± 0.82	9.53 ± 2.78	13.17 ± 4.23	14.90 ± 4.65
α-ABA	13.32 ± 3.03^a^	3.68 ± 0.87^b^	3.03 ± 0.71^b^	2.60 ± 0.27^b^
Valine	49.50 ± 6.54	40.00 ± 10.26	37.67 ± 7.88	35.67 ± 3.18
Cystine	5.61 ± 0.18	ND	ND	ND
Methionine	5.34 ± 1.59	1.73 ± 0.32	1.90 ± 0.20	1.47 ± 0.37
Cystathionine	37.66 ± 12.00^a^	1.88 ± 0.48^b^	2.97 ± 0.36^b^	1.78 ± 0.30^b^
Isoleucine	28.50 ± 5.39	19.67 ± 5.36	20.33 ± 3.28	19.33 ± 0.88
Leucine	37.50 ± 5.74	28.00 ± 7.77	27.33 ± 4.81	27.33 ± 1.45
Tyrosine	3.68 ± 1.51	1.97 ± 0.26	5.17 ± 2.41	4.33 ± 1.96
Phenylalanine	29.00 ± 4.04^a^	18.33 ± 1.86^b^	19.67 ± 1.86^b^	13.67 ± 0.88^b^
β-Alanine	15.47 ± 0.33^a^	5.76 ± 1.71^b^	9.98 ± 3.23^a^^b^	6.04 ± 2.21^b^
Tryptophan	6.63 ± 0.72^a^	2.53 ± 0.38^b^	2.77 ± 0.09^b^	1.73 ± 0.30^b^
Ornithine	34.67 ± 4.33^a^	14.33 ± 3.38^b^	25.67 ± 8.84^a^^b^	17.67 ± 4.26^a^^b^
Lysine	65.67 ± 6.96^a^	42.67 ± 12.33^a^^b^	41.00 ± 7.02^a^^b^	36.00 ± 1.16^b^
Histidine	55.25 ± 17.05	32.33 ± 3.38	32.33 ± 2.91	25.67 ± 6.69
Arginine	272.25 ± 58.23^b^	223.67 ± 21.94^b^	439.67 ± 4.67^a^	276.33 ± 37.33^b^
TFAAs	3993.00 ± 361.73^a^	2955.43 ± 129.94^b^	2875.62 ± 142.01^b^	2595.69 ± 174.48^b^

Data are presented as mean ± SE.

^‘a, b’^: different lowercase letters at the same row means statistical difference between crabs of all experimental treatments with *P* < 0.05. Free amino acid contents < 5 nmol mL ^-1^ are not listed in this table. TFAAs: total free amino acids; α-ABA: α- amino-n-butyric acid; α-AAA: α-aminoadipicacid; ND: value not detected.

### Gill Na^+^/K^+^-ATPase activity and its mRNA expression

**Fig 3:**
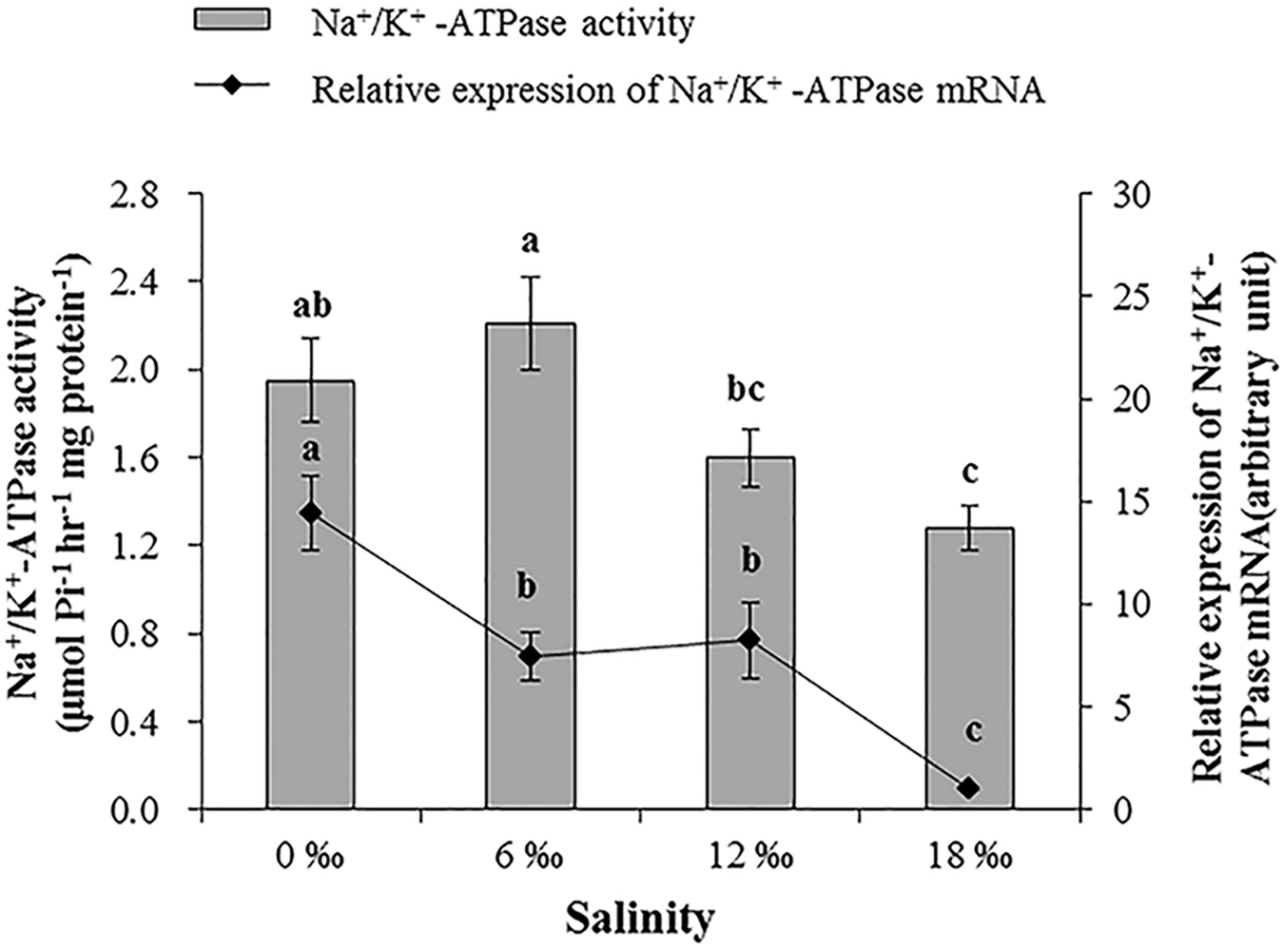
The mean gill Na^+^/K^+^-ATPase activity (μmol Pi h^-1^ mg protein^-1^) and its mRNA expression level (± SE) in posterior gills of adult male *E*. *sinensis* subjected to different salinities.

A decreasing trend was found for the activity of Na^+^ /K^+^-ATPase and its mRNA expression level in the posterior gills with increasing salinity (*P* < 0.05, Fig 3). The highest activity of Na^+^/K^+^-ATPase was found for the 6 ‰ treatment, which was significantly higher than those of the 12 ‰ and 18 ‰ treatments (*P* < 0.05), but not that of the 0 ‰ treatment (*P* > 0.05). The highest and the lowest mRNA expression level of Na^+^/K^+^-ATPase were found for the 0 ‰ and 18 ‰ treatments, respectively (*P* < 0.05).

### Gill fatty acid profile

The fatty acids profile in the anterior gill was shown in Table 3. For saturated fatty acid (SFA), the C18:0 and total saturated fatty acids (∑SFA) showed an overall decreasing trend with increasing salinity (*P* < 0.05), while no significant changes were found for the other SFAs. For monounsaturated fatty acid (MUFA), the percentages of C16:1n7, C18:1n7 and total monounsaturated fatty acids (∑MUFA) firstly increased significantly with increasing salinity to reach a peak at 6 ‰ but then decreased with increasing salinity (*P* < 0.05). For polyunsaturated fatty acid (PUFA), the 6 ‰ also resulted in the highest percentage of C18:3n3, while the C20:5n3 decreased with increasing salinity (*P* < 0.05). The C22:6n3 showed an increasing trend with increasing salinity while the highest and the lowest n-3/n-6 ratio was found for the 12 ‰ and 18 ‰ treatments, respectively (*P* < 0.05).

**Table 3:** Fatty acids profile (% of total fatty acids) in the anterior gills of adult male *E*. *sinensis* subjected to different salinities.

Fatty acids	Salinity			
	0 ‰	6 ‰	12 ‰	18 ‰
C14:0	0.36 ± 0.04	0.30 ± 0.04	0.31 ± 0.06	0.33 ± 0.06
C15:0	0.28 ± 0.03	0.24 ± 0.01	0.25 ± 0.01	0.25 ± 0.01
C16:0	14.23 ± 0.63	13.39 ± 0.27	13.33 ± 0.20	13.07 ± 0.50
C17:0	0.43 ± 0.03	0.43 ± 0.05	0.43 ± 0.08	0.39 ± 0.03
C18:0	7.45 ± 0.05^a^^b^	7.54 ± 0.24^a^	7.16 ± 0.16^a^^b^	6.69 ± 0.25^b^
C20:0	0.74 ± 0.04	0.71 ± 0.01	0.69 ± 0.02	0.69 ± 0.04
C22:0	0.74 ± 0.01	0.73 ± 0.03	0.73 ± 0.01	0.68 ± 0.03
∑SFA	24.23 ± 0.64^a^	23.32 ± 0.57^a^^b^	22.88 ± 0.16^a^^b^	22.09 ± 0.23^b^
C16:1n7	2.79 ± 0.09^b^	3.14 ± 0.08^a^	2.51 ± 0.02^b^	2.55 ± 0.09^b^
C18:1n9	18.17 ± 0.13	17.76 ± 0.22	18.07 ± 0.09	17.99 ±0.03
C18:1n7	2.94 ± 0.02^c^	3.56 ± 0.06^a^	3.42 ± 0.06^a^^b^	3.23 ± 0.11^b^
C20:1n7	0.83 ± 0.03	0.85 ± 0.01	0.87 ± 0.01	0.78 ± 0.06
∑MUFA	24.72 ± 0.26^a^^b^	25.30 ± 0.18^a^	24.87 ± 0.03^a^^b^	24.54 ± 0.11^b^
C18:2n6	7.26 ± 0.04	6.75 ± 0.39	7.34 ± 0.07	7.10 ± 0.17
C18:3n3	0.57 ± 0.04^b^	0.71 ± 0.01^a^	0.61 ± 0.01^b^	0.43 ± 0.01^c^
C20:2n6	2.28 ± 0.08	2.36 ± 0.02	2.21 ± 0.06	2.23 ± 0.10
C20:3n6	0.18 ± 0.02	0.18 ± 0.02	0.22 ± 0.00	0.20 ± 0.00
C20:4n6	16.43 ± 0.12	17.17 ± 0.00	17.33 ± 0.09	15.86 ± 0.86
C20:5n3	11.40 ± 0.06^a^	10.59 ± 0.19^a^^b^	10.41 ± 0.22^b^	10.29 ± 0.40^b^
C22:6n3	5.13 ± 0.02^b^	5.74 ± 0.10^a^^b^	5.64 ± 0.06^a^^b^	6.26 ± 0.32^a^
∑PUFA	43.24 ± 0.13	43.49 ± 0.09	43.74 ± 0.49	42.35 ± 1.84
∑n-3PUFA	17.10 ± 0.01	17.03 ± 0.27	16.65 ± 0.28	16.97 ± 0.72
∑n-6PUFA	26.15 ± 0.14	26.46 ± 0.36	27.09 ± 0.21	25.38 ± 1.12
n-3/n-6	0.66 ± 0.01^a^	0.65 ± 0.02^a^^b^	0.62 ± 0.01^b^	0.67 ± 0.00^a^
∑HUFA	33.14 ± 0.06	33.67 ± 0.30	33.59 ± 0.36	32.60 ± 1.57

Data are presented as mean ± SE.

^‘a, b, c’^: different lowercase letters at the same row means statistical difference between crabs of all experimental treatments with *P* < 0.05. ∑SFA: total saturated fatty acid; ∑MUFA: total monounsaturated fatty acid; ∑PUFA: total polyunsaturated fatty acid; ∑HUFA: total highly unsaturated fatty acid.

For fatty acid profile in the posterior gill (Table 4). The percentage of C18: 0 firstly increased slightly with salinity to reach a peak at 6 ‰ but then decreased significantly with salinity (*P* < 0.05). For MUFA, 6 ‰ treatment had the highest percentages of C18: 1n9 and ∑MUFA, while the C18:1n7 percentage firstly showed an increasing trend with salinity with a peak detected for the 12 ‰ treatment (*P* < 0.05) before decreased slightly at 18 ‰ (*P* > 0.05). For PUFA, the highest percentages of C18:2n6 and C18:3n3 was found for 6 ‰ treatment (*P* < 0.05), however, the C20: 2n6, C20:5n3, ∑n-3 PUFA and n-3/n-6 ratio decreased with increasing salinity. The percentages of C20:4n6 and ∑n-6 PUFA showed an increasing trend with salinity (*P* < 0.05); the ∑PUFA firstly decreased and then increased significantly with increasing salinity, and the lowest level was detected for 6 ‰ treatment (*P* < 0.05).

**Table 4:** Fatty acids profile (% of total fatty acids) in the posterior gills of adult male *E*. *sinensis* subjected to different salinities.

Fatty acids	Salinity			
	0 ‰	6 ‰	12 ‰	18 ‰
C14:0	0.22 ± 0.03	0.22 ± 0.02	0.24 ± 0.00	0.23 ± 0.02
C15:0	0.20 ± 0.02	0.23 ± 0.01	0.21 ± 0.00	0.22 ± 0.00
C16:0	12.22 ± 0.76	14.16 ± 0.58	12.60 ± 0.20	12.38 ± 0.31
C17:0	0.38 ± 0.02	0.37 ± 0.01	0.45 ± 0.08	0.46 ± 0.05
C18:0	8.03 ± 0.07^a^	8.11 ± 0.13^a^	7.49 ± 0.11^b^	7.47 ± 0.07^b^
C20:0	0.70 ± 0.05	0.62 ± 0.01	0.67 ± 0.01	0.63 ± 0.02
C22:0	0.75 ± 0.06^a^	0.53 ± 0.03^b^	0.64 ± 0.04^a^^b^	0.72 ± 0.01^a^
∑SFA	22.48 ± 0.65	24.23 ± 0.69	22.29 ± 0.43	22.10 ± 0.47
C16:1n7	2.19 ± 0.19	2.66 ± 0.26	2.53 ± 0.06	2.35 ± 0.02
C18:1n9	19.53 ± 0.47^b^	21.60 ± 0.18^a^	20.97 ± 0.19^a^	20.70 ± 0.17^a^
C18:1n7	2.91 ± 0.05^b^	3.24 ± 0.06^a^	3.25 ± 0.01^a^	3.16 ± 0.08^a^
C20:1n7	0.78 ± 0.01	0.74 ± 0.01	0.81 ± 0.02	0.82 ± 0.06
∑MUFA	23.22 ± 0.51^b^	25.57 ± 0.22^a^	25.02 ± 0.17^a^	24.67 ± 0.04^a^
C18:2n6	8.12 ± 0.21^b^	8.73 ± 0.17^a^	8.07 ± 0.11^b^	7.72 ± 0.04^b^
C18:3n3	0.56 ± 0.04^a^	0.61 ± 0.01^a^	0.58 ± 0.04^a^	0.45 ± 0.01^b^
C20:2n6	3.33 ± 0.12^a^	2.92 ± 0.11^b^	2.76 ± 0.03^b^	2.59 ± 0.07^b^
C20:3n6	0.14 ± 0.02	0.17 ± 0.03	0.19 ± 0.02	0.21 ± 0.01
C20:4n6	13.71 ± 0.72^b^	13.89 ± 0.10^b^	15.19 ± 0.15^a^^b^	16.46 ± 0.11^a^
C20:5n3	15.76 ± 0.34^a^	12.67 ± 0.11^b^	12.32 ± 0.12^b^^c^	11.53 ± 0.20^c^
C22:6n3	5.69 ± 0.54	5.16 ± 0.06	5.70 ± 0.20	6.16 ± 0.13
∑PUFA	47.29 ± 1.48^a^	44.13 ± 0.04^b^	44.80 ± 0.36^a^^b^	45.10 ± 0.13^a^^b^
∑n-3PUFA	22.00 ± 0.83^a^	18.43 ± 0.05^b^	18.60 ± 0.28^b^	18.13 ± 0.33^b^
∑n-6PUFA	25.29 ± 0.65^b^	25.70 ± 0.01^b^	26.20 ± 0.08^a^^b^	26.97 ± 0.20^a^
n-3/n-6	0.87 ± 0.01^a^	0.72 ± 0.01^b^	0.71 ± 0.01^b^	0.68 ± 0.02^b^
∑HUFA	35.29 ± 1.61	31.88 ± 0.03	33.40 ± 0.49	34.36 ± 0.23

Data are presented as mean ± SE.

^‘a, b, c’^: different lowercase letters at the same row means statistical difference between crabs of all experimental treatments with *P* < 0.05. ∑SFA: total saturated fatty acid; ∑MUFA: total monounsaturated fatty acid; ∑PUFA: total polyunsaturated fatty acid; ∑HUFA: total highly unsaturated fatty acid.

### Hemolymph metabolism indices

The contents of GLU and UA decreased significantly with increasing salinity while an increasing trend was found for TG (*P* < 0.05, Table 5). On the other hand, HDL-C and MDA showed firstly an increasing trend with increasing salinity with a peak at 12 ‰ before decreased at 18 ‰ (*P* < 0.05). Meanwhile, the highest and the lowest activities of γ-GT and ALP were found for the 6 ‰ and 18 ‰ treatments, respectively (*P* < 0.05). No significant difference was detected for TC, LDL-C, urea, SOD and ACP among all treatments (*P* > 0.05) (Table 5).

**Table 5:** Hemolymph metabolism indices of adult male *E*. *sinensis* subjected to different salinities.

Indices	Salinity			
	0 ‰	6 ‰	12 ‰	18 ‰
GLU (mmol L^-1^)	6.88 ± 0.87^a^	6.07 ± 0.55^a^	3.44 ± 0.26^b^	3.59 ± 0.31^b^
TC (mmol L^-1^)	0.59 ± 0.10	0.65 ± 0.07	0.70 ± 0.08	0.65 ± 0.06
TG (mmol L ^-1^)	0.14 ± 0.01^b^	0.17 ± 0.01^a^^b^	0.17 ± 0.01^a^^b^	0.19 ± 0.02^a^
HDL-C (mmol L^-1^)	0.23 ± 0.04^b^	0.28 ± 0.03^a^^b^	0.33 ± 0.03^a^	0.28 ± 0.02^a^^b^
LDL-C (mmol L^-1^)	0.19 ± 0.03	0.20 ± 0.02	0.19 ± 0.02	0.21 ± 0.02
UA (mmol L^-1^)	24.54 ± 5.99^a^	23.98 ± 3.42^a^	19.67 ± 3.07^a^^b^	9.30 ± 1.12^b^
Urea (μmol L^-1^)	2.22 ± 0.24	1.89 ± 0.13	2.26 ± 0.07	1.92 ± 0.09
SOD (U L^-1^)	86.31 ± 1.18	84.69 ± 1.61	84.22 ± 1.89	85.52 ± 1.04
MDA (nmol mL ^-1^)	14.02 ± 1.37^b^	18.17 ± 1.29^a^^b^	20.69 ± 1.94^a^	17.79 ± 0.98^a^^b^
γ-GT (U L^-1^)	0.87 ± 0.04^b^^c^	1.19 ± 0.08^a^	1.08 ± 0.09^a^^b^	0.76 ± 0.07^c^
ALP (U L^-1^)	4.86 ± 0.36^a^	5.53 ± 0.71^a^	5.09 ± 0.45^a^	2.91 ± 0.20^b^
ACP (U 100 mL ^-1^)	0.98 ± 0.14	0.97 ± 0.09	0.92 ± 0.08	1.07 ± 0.16

Data are presented as mean ± SE.

^‘a, b, c’^: different lowercase letters at the same row means statistical difference between crabs of all experimental treatments with *P* < 0.05. GLU: glucose; TC: total cholesterol; TG: triglyceride; HDL-C: high-density lipoprotein cholesterol; LDL-C: low-density lipoprotein cholesterol; UA: uric acid; SOD: superoxide dismutase; MDA: malonaldehyde; γ-GT: γ- glutamyl transferase; ALP: alkaline phosphatase; ACP: acid phosphatase.

### Hepatopancreas metabolism indices

The total lipids in hepatopancreas firstly showed an increasing trend with increasing salinity, with a peak detected for the 12 ‰ treatment, before decreased at 18 ‰ (*P* < 0.05, Table 6). Urea increased significantly with increasing salinity (*P* < 0.05) while the activity of γ-GT firstly increased significantly with salinity to reach a peak at 12 ‰ (*P* < 0.05) but decreased slightly 18 ‰. SOD showed a fluctuating pattern with the highest level detected for the 18 ‰ treatment while the highest activity of ALP was found for the 6 ‰ treatment (*P* < 0.05). No significant difference was detected for GLU, UA and MDA as well as the activity of ACP among treatments (*P >* 0.05).

**Table 6:** Hepatopancreas metabolism indices of adult male *E*. *sinensis* subjected to different salinities.

Indices	Salinity			
	0 ‰	6 ‰	12 ‰	18 ‰
Lipid (% wet weight)	31.93 ± 1.72^b^	37.94 ± 2.35^a^^b^	40.59 ± 2.70^a^	35.74 ± 1.48^a^^b^
GLU (mmol g ^-1^ tissue)	49.56 ± 3.28	47.68 ± 4.77	48.83 ± 4.80	45.38 ± 3.46
UA (mmol g ^-1^ protein)	1.65 ± 0.15	1.71 ± 0.33	1.63 ± 0.33	1.25 ± 0.21
Urea (μmol g ^-1^ protein)	0.29 ± 0.02^b^	0.28 ± 0.02^b^	0.31 ± 0.02^a^^b^	0.34 ± 0.02^a^
SOD (U g ^-1^ protein)	29.81 ± 1.79^b^	32.95 ± 2.57^a^^b^	31.46 ± 1.36^a^^b^	36.92 ± 2.33^a^
MDA (n mol g ^-1^ protein)	2.70 ± 0.12	2.65 ± 0.24	2.95 ± 0.23	3.03 ± 0.20
γ-GT (U g ^-1^ protein)	2.72 ± 0.30^b^	3.05 ± 0.58^b^	5.41 ± 0.54^a^	4.69 ± 1.01^a^^b^
ALP (U g ^-1^ protein)	6.51 ± 0.71^b^^c^	8.36 ± 0.35^a^	7.98 ± 0.33^a^^b^	5.80 ± 0.80^c^
ACP (U 100g ^-1^ protein)	2.54 ± 0.27	2.90 ± 0.27	3.05 ± 0.24	3.32 ± 0.37

Data are presented as mean ± SE.

^‘a, b, c’^: different lowercase letters at the same row means statistical difference between crabs of all experimental treatments with *P* < 0.05. GLU: glucose; UA: uric acid; SOD: superoxide dismutase; MDA: malonaldehyde; γ-GT: γ- glutamyl transferase; ALP: alkaline phosphatase; ACP: acid phosphatase.

## Discussion

Past studies on salinity effects on gonadal development of crustaceans have been focused on females of various shrimp species [34–35], so far little attention have been paid to male crustaceans [14]. The present study showed that the GSI of adult male *E*. *sinensis* increased significantly at salinity of 12 ‰ and 18 ‰ as compared to lower salinities of 0 ‰ and 6 ‰, suggesting that brackish water promotes gonadal development of the adult *E*. *sinensis*. Possible explanations for such an effect include: 1) the osmolality of brackish water is closer to that in the hemolymph of the male *E*. *sinensis*, hence reduced energy consumption on osmoregulation [10, 36], resulting in more nutrients/energy being channeled to gonadal development [37]; 2) certain ions, such as calcium, are substantially higher in brackish water, which may have the function in stimulating gonadal development of crustaceans [14, 38]. Previous studies have also shown that ovarian development of several marine crustaceans were delayed at low salinities [39–40], which might be due to these crustaceans having to spend much of energy for osmoregulation, hence leading to poor gonadal development [40–41].

Gills play a key role in osmotic and ionic regulation in crustaceans [9, 42]. Previous studies have demonstrated that the anterior and posterior gills were specialized for respiration and osmoregulation, respectively, in crabs [25, 43]. The function of posterior gills on osmotic and ionic regulation is dependent on enzymes for ionic transportation, in particular Na^+^/K^+^-ATPase, and transport proteins [9, 26]. In the current study, the activity of Na^+^/K^+^-ATPase and its mRNA expression level in the posterior gills decreased significantly with increasing salinity, similar results was shown for *E*. *sinensis* subjected to short-term salinity changes [44–45] as well as in several marine crabs [46–47]. Such a result could be explained by the fact that at higher salinities, particularly 18 ‰, osmolality of hemolymph is very similar to that of external media than in the case of freshwater (e.g. at 18 ‰, hemolymph osmolality/external osmolality = 659.43 ± 9.34/538.00 ± 2.00 mOsm kg H_2_O ^-1^; at 0 ‰, hemolymph osmolality/external osmolality = 608.33 ± 19.54/10.67 ± 0.58 mOsm kg H_2_O ^-1^, S1 Fig), hence the Na^+^ / K+—ATPase was reduced as salinity increased.

Previous studies have shown that crustaceans, including crabs, responded to ambient salinity changes by changing fatty acids compositions in different tissues, particularly in gills [22, 47–48]. Similar to the result reported for the mud crab (*Scylla serrata*) [22], the percentages of C18:2n6, C20:2n6, C20:5n3, as well as ∑PUFA and ∑n-3PUFA in the posterior gills of male *E*. *sinensis* decreased significantly with elevated salinity they exposed to. Such a result might be an adaptive response to reduce membrane fluidity and as the result, lower gill Na^+^/K^+^-ATPase activity and energy were required to maintain ionic concentrations at higher salinities [22, 49].

*E*. *sinensis* is a euryhaline species, but is also known as a strong hyper- osmoregulator but a weak hypo-osmoregulator [9, 45]. In this study, the hemolymph osmolality and ionic concentrations showed an overall increasing trend with increasing salinity, and were always higher than those in the external media except for K^+^ and Mg^2+^. Previous studies showed that Na^+^ and Cl^-^ were two most important ions contribute to hemolymph osmolality of crustaceans [15–16]. In this study, the concentrations of Na^+^ and Cl^-^ were several orders higher than other ions, and similar results were reported for the mud crabs, *Scylla paramamosain* [50] and *S*. *serrata* [22], and the blue swimmer crab *Portunus pelagicus* [51]. Additionally, the concentrations of K^+^ and Mg^2+^ increased significantly with increasing salinity, which may be explained by 1) K^+^ and Mg^2+^ also contributed to hemolymph osmolality although relatively minor; 2) high Mg^2+^ level in hemolymph could inhibit Na^+^/K^+^-ATPase activity [25, 52], which might related to decreased activity of Na^+^/K^+^-ATPase found in the posterior gills under higher salinities.

In addition to inorganic ions, the hemolymph free amino acids (FAAs) are also known as important contributors to osmoregulation, which play an important role in intracellular osmoregulation in crustaceans although their concentrations are lower than those of inorganic ions [17–18]. The major FAAs in hemolymph of the male *E*. *sinensis* were found to be taurine, glutamine, proline, glycine, alanine and arginine, ranging from 200 to 1200 nmol mL^-1^. A decreasing trend was found for threonine, proline, phenylalanine, tryptophan, lysine and total free amino acids (TFAAs) with increasing salinity, which is in agreement with a previous study [53]. A possible explanation for such a result could be that due to substantially bigger differences between osmolality of the hemolymph and external media at low salinities, the hemolymph protein was catabolized into FAAs to facilitate osmoregulation under such hypo-osmotic condition [17, 53]. Therefore some specific FAAs, in particular proline, alanine, glycine, glutamine, taurine and arginine, may acted as the osmoeffectors for the male *E*. *sinensis* subjected to long-term higher salinity exposure. However the biochemical pathways leading to changes in FAAs during higher salinity acclimation remain to be studied.

The biochemical parameters generally mirror the physiological status of *E*. *sinensis* subjected to different salinities [10, 15]. In the present study, the content of GLU in the hemolymph was found decreasing significantly with increasing salinity. GLU is a major energy source for osmoregulation, which could be enhanced by elevated catabolism rates of glycogen into monosaccharide under hypo-osmotic environment [54–55]. Since osmolality of brackish water is closer to that of hemolymph when compared to freshwater; relatively fewer energy was required for osmoregulation by the crabs kept in brackish water [10], hence less GLU was produced by them. Moreover, lipid is also an important energy source for metabolism of *E*. *sinensis* [56], which could provide the energy needed for the crabs to cope with osmotic stresses [57]. In this study, the lowest content of hemolymph TG was found for the 0 ‰ treatment, which may be a consequence that more TG was decomposed to provide energy for active ion transportation under such condition [58].

The cholesterol is not only an important component of cell membranes, but also a precursor for biosynthesis hormones regulating gonadal development and molt in crustaceans [38, 59]. The hemolymph TC and HDL-C increased significantly with increasing salinity and peaked at 12 ‰, which may be related to increasing cholesterol requirement for gonadal development for the crabs in brackish water [14]. Meanwhile, the tissues content of urea, a metabolite of protein, is believed to directly reflect protein metabolism [60–61]. In the present study, an increasing trend was found for urea in the hepatopancreas with the increasing salinity, a similar result was observed in the Kuruma shrimp *Marsupenaeus japonicas* [62]. Such results may reflect decreasing ammonia excretion rate of the male *E*. *sinensis* in brackish water. Furthermore, the UA as the end product of nitrogen metabolism [63] decreased with increasing salinity in both hemolymph and hepatopancreas, which may be a result of increased decomposition of UA into urea at higher salinities [63–64] and consistent with the result of higher hepatopancreas urea contents at such salinities. These results together indicated that the male *E*. *sinensis* had relatively low protein catabolism rate when in brackish water.

SOD is an important antioxidant enzyme of the antioxidant defense systems in crustaceans, which can scavenge the free radicals and prevents tissue damage [12, 65]. The present study detected significant increase of SOD with increasing salinity and the highest activity of SOD in the hepatopancreas was found at 18 ‰, similar result was reported for the ridgetail white prawn *Exopalaemon carinicauda* [66]. The result indicated higher levels of free radicals were probably found in the tissues of the male crabs in brackish water, therefore the antioxidant system was enhanced to scavenge the free radicals [65, 67]. Meanwhile, MDA is the product of lipid peroxidation [15]. In this study, both the pattern of in hemolymph MDA level changed with salinity and the highest MDA detected at 12 ‰ were consistent with those of lipid content changes in hepatopancreas. Such a pattern suggests that probably more lipids were consumed for osmotic regulation at low salinities [57]. The γ-GT is a plasma membrane glycoprotein, which plays important roles in glutathione metabolism, amino acid transportation and detoxification [68–69]. The present study showed that the activity of γ-GT in the hepatopancreas was the highest for the 12 ‰ treatment, indicating 12 ‰ might represent as an optimal salinity for fast gonadal development of the male *E*. *sinensis*.

ACP and ALP are two important phosphatases that are crucial for metabolism and the immune system of *E*. *sinensis* [15]. In the current study, the highest activity of ALP in hemolymph and hepatopancreas was both found for the 6 ‰ treatment, with ALP of the 12 ‰ treatment followed closely and not significantly different, indicating appropriate salinity could enhance immune responses of the male *E*. *sinensis*. A similar result was reported for the euryhaline crab *Chasmagnathus granulatus* [70].

## Conclusion

The present study demonstrated that brackish water promoted gonadal development of the adult male *E*. *sinensis*. The brackish water conditions increased hemolymph osmolality and major ion concentrations while reduced Na^+^/K^+^-ATPase activity and its mRNA expression levels, as well as total polyunsaturated fatty acids contents in the posterior gills. Consequently the adult male *E*. *sinensis* subjected to salinity of 12 ‰ and 18 ‰ likely had lower energy expenditures on osmoregulation, which could be channeled for gonadal development.

## Supporting information

S1 Fig The mean hemolymph osmolality of adult male *E*. *sinensis* and external osmolality (mOsm kg H_2_O ^-1^) form different salinity treatments. Different lowercase letters “a, b” on the tops of the bars indicated significant differences (*P* < 0.05) in hemolymph osmolality among different salinity treatments, while the different capital letters “A, B, C, D” indicated significant differences (*P* < 0.05) in external osmolality among all different salinity treatments. The superscript ‘**’ on the top of the bars indicated vary significant differences (*P* < 0.01) on the osmolality between the hemolymph of male crabs and their respectively external media.
(TIF)

## References

[1] Sui LY, Zhang FM, Wang XM, Bossier P, Sorgeloos P, Hänfling B. Genetic diversity and population structure of the Chinese mitten crab *Eriocheir sinensis* in its native range. Marine Biology. 2009; 156(8): 1573–1583. doi: 10.1007/s00227-009-1193-2

[2] Cheng YX, Wu XG, Yang XZ, Hines AH. Current trends in hatchery techniques and stock enhancement for Chinese mitten crab, *Eriocheir japonica sinensis*. Reviews in Fisheries Science. 2008; 16(1–3): 377–386. doi: 10.1080/10641260701681698

[3] Zhang TL, Li ZJ, Cui YB. Survival, growth, sex ratio, and maturity of the Chinese mitten crab (*Eriocheir sinensis*) reared in a Chinese pond. Journal of Freshwater Ecology. 2001; 16(4): 633–640. doi: 10.1080/02705060.2001.9663855

[4] Bentley MG. The global spread of the Chinese mitten crab *Eriocheir sinensis*. In the Wrong Place—Alien Marine Crustaceans: Distribution, Biology and Impacts Invading Nature—Springer Series in Invasion Ecology, Part 2. 2011; 6: 107–127.

[5] Zhang LS. The crabs life history research and juvenile crab fishing. Aquatic Science and Technology Intelligence. 1973; 1(2): 5–21 (in Chinese).

[6] Herborg LM, Rushton SP, Clarea AS, Bentley MG. Spread of the Chinese mitten crab (*Eriocheir sinensis* H. Milne Edwards) in Continental Europe: analysis of a historical data set. Hydrobiologia. 2003; 503(1–3): 21–28. doi: 10.1023/B:HYDR.0000008483.63314.3c

[7] Wei W, Wu JM, Wei H. Physiological mechanism of precociousness influenced by salinity in juvenile *Eriocheir sinensis*. Journal of Fishery Sciences of China. 2007; 14(2): 275–280 (in Chinese with English abstract).

[8] Rathmayer M, Siebers D. Ionic balance in the freshwater-adapted Chinese crab, *Eriocheir sinensis*. Journal of Comparative Physiology B. 2001; 171(4): 271–281. doi: 10.1007/s003600100173 11409624 10.1007/s003600100173

[9] Wang RF, Zhuang P, Feng GP, Zhang LZ, Huang XR, Jia XY. Osmotic and ionic regulation and Na^+^/K^+^-ATPase, carbonic anhydrase activities in mature Chinese mitten crab, *Eriocheir sinensis* H. Milne Edwards, 1853 (Decapoda, Brachyura) exposed to different salinities. Crustaceana. 2012; 85(12–13): 1431–1447. doi: 10.1163/15685403-00003125

[10] Jia XY, Zhuang P, Feng GP, Wang RF, Lu J, Huang XR. The relationship between hemolymph biochemical parameters and salinity in female parent Chinese mitten crab (*Eriocheir sinensis*). Journal of Fisheries of China. 2012; 36(1): 91–97 (in Chinese with English abstract). doi: 10.3724/SP.J.1231.2012.27614

[11] Péqueux A, Gilles R. The transepithelial potential difference of isolated perfused gills of the Chinese crab *Eriocheir sinensis* acclimated to fresh water. Comparative Biochemistry and Physiology, Part A: Physiology. 1988; 89(2): 163–172. doi: 10.1016/0300-9629(88)91074-2

[12] Lu J, Zhuang P, Feng GP, Zhang LZ, Wang RF. Response of osmoregulation and antioxidation system to water salinity in Parent Chinese mitten crab (*Eriocheir sinensis*). Marine Fisheries. 2011; 33(1): 39–45 (in Chinese with English abstract). doi: 10.13233/j.cnki.mar.fish.2011.01.013

[13] Wang SC, Xu L. Changes in serum total protein and hemocyanin content of *Eriocheir sinensis* subjected different salinity. Journal of Huainan Teachers College. 2003; 5(3): 24–26 (in Chinese).

[14] Wu XG, Zhao YT, He J, Huang Q, Huang ZF, Liu H, et al Effect of brackish water and fresh water on gonadal development and mating behavior in adult Chinese mitten crab. Chinese Journal of Zoology. 2013; 48(3): 99–105 (in Chinese with English abstract). doi: 10.13859/j.cjz.2013.04.008

[15] Zhao L, Long XW, Wu XG, He J, Shi YH, Zhang GY, et al Effects of water aslinity on osmoregulation and physiological metabolism of adult male Chinese mitten crab *Eriocheir sinensis*. Acta Hydrobiologica Sinica. 2016; 40(1): 27–34 (in Chinese with English abstract). doi: 10.7541/2016.4

[16] Chen JC, Chia PG. Osmotic and ionic concentrations of *Scylla Serrata* (Forskal) subjected to different salinity levels. Comparative Biochemistry and Physiology, Part A: Molecular and Integrative Physiology. 1997; 177(2): 239–244. doi: 10.1016/S0300-9629(96)00237-X

[17] Huong DTT, Yang WJ, Okuno A, Wilder MN. Changes in free amino acids in the hemolymph of giant freshwater prawn *Macrobrachium rosenbergii* exposed to varying salinities: relationship to osmoregulatory ability. Comparative Biochemistry and Physiology, Part A: Molecular and Integrative Physiology. 2001; 128(2): 317–326. doi: 10.1016/S1095-6433(00)00310-X 11223393 10.1016/s1095-6433(00)00310-x

[18] Abe H, Okuma E, Amano H, Noda H, Watanabe K. Effects of seawater acclimation on the levels of free D- and L- alanine and other osmolytes in the Japanese mitten crab *Eriocheir japonicus*. Fisheries science. 1999; 65(6): 949–954.

[19] Genovese G, Luchetti CG, Luquet CM. Na^+^/K^+^-ATPase activity and gill ultrastructure in the hyper-hypo-regulating crab *Chasmagnathus granulatus* acclimated to dilute, normal and concentrated seawater. Marine Biology. 2004; 144(1): 111–118. doi: 10.1007/s00227-003-1169-6

[20] Henry RP, Lucu C, Onken H, Weihrauch D. Multiple functions of the crustacean gill: Osmotic/ionic regulation, acid-base balance, ammonia excretion and bioaccumulation of toxic metals. Frontiers in Physiology. 2012; 3:1–33. doi: 10.3389/fphys.2012.00431 23162474 10.3389/fphys.2012.00431 PMC3498741

[21] Haines TH. Water transport across biological membranes. FEBS Letters. 1994; 346(1):115–122. doi.org/10.1016/0014-5793(94)00470-6 8206149 10.1016/0014-5793(94)00470-6

[22] Romano N, Wu XG, Zeng CS, Genodepa J, Ellman J. Growth, osmoregulatory responses and changes to the lipid and fatty acid composition of organs from the mud crab, *Scylla serrata*, over abroad salinity range. Marine Biology Research. 2014; 10(5): 460–471. doi: 10.1080/17451000.2013.819981

[23] Zhang LS, Li J. Aquaculture Technology of *Eriocheir sinensis*. Beijing: Golden Shield Press; 2002 (in Chinese).

[24] Lai W. The habits and reproductive migratory of *Eriocheir sinensis*. Fish World. 1994; (6): 33–41(in Chinese).

[25] Lü F, Pan LQ, Ren JY. A study on the characteristics of gill Na-K-ATPase of the *Eriocheir sinensis*. Transact ions of Oceanology and Limnology. 2004; (3): 47–53 (in Chinese with English abstract). doi: 10.13984/j.cnki.cn37-1141.2004.03.008

[26] Henry RP, Gehnrich S, Weihrauch D, Towle DW. Salinity-mediated carbonic anhydrase induction in the gills of the euryhaline green crab, *Carcinus maenas*. Comparative Biochemistry and Physiology, Part A: Molecular and Integrative Physiology. 2003; 136(2): 243–258. doi: 10.1016/S1095-6433(03)00113-2 14511744 10.1016/s1095-6433(03)00113-2

[27] Chew SF, Ho SY, Ip YK. Free amino acids and osmoregulation in the intertidal pulmonate *Onchidium tumidium*. Marine Biology. 1999; 134(4): 735–741. doi: 10.1007/s002270050590

[28] Guo ZH, Yang ZG, Cheng YX, Ji LY, Que YQ, Liu ZW, et al Molecular characterization, tissue expression of acyl-CoA Δ9-desaturaselike gene, and effects of dietary lipid levels on its expression in the hepatopancreas of the Chinese mitten crab (*Eriocheir sinensis*). Aquaculture. 2013; 402–403: 58–65. doi: 10.1016/j.aquaculture.2013.03.033

[29] Folch J, Lees M, Sloane-Stanley GH. A simple method for the isolation and purification of total lipides from animal tissues. Journal of Biological Chemistry. 1957; 226: 497–509. 13428781

[30] Morrison WR, Smith LM. Preparation of fatty acid methyl esters and dimethylacetals from lipids with boron flouride- methanol. Journal of Lipid Research. 1964; 5: 600–608. 14221106

[31] Qiu RJ, Cheng YX, Huang XX, Wu XG, Yang XZ, Tong R. Effect of hypoxia on immunological, physiological response, and hepatopancreatic metabolism of juvenile Chinese mitten crab *Eriocheir sinensis*. Aquaculture International. 2011; 19(2): 283–299. doi: 10.1007/s10499-010-9390-z

[32] Deng JM, An QC, Bi BL, Wang QJ, Kong LF, Tao LL, et al Effect of ethanolic extract of propolis on growth performance and plasma biochemical parameters of rainbow trout (*Oncorhynchus mykiss*). Fish Physiology and Biochemistry. 2011; 37(4): 959–967. doi: 10.1007/s10695-011-9493-0 21559799 10.1007/s10695-011-9493-0

[33] Ohkawa H, Ohishi N, Yagi K. Assay for lipid peroxides in animal tissues by thiobarbituric acid reaction. Analytical Biochemistry. 1979; 95: 351–358. 36810 10.1016/0003-2697(79)90738-3

[34] Gelin A, Crivelli AJ, Rosecchi E, Kerambrum P. Can salinity changes affect reproductive success in the brown shrimp *Crangon crangon*? Journal of Crustacean Biology. 2001; 21(4): 905–911. doi: 10.1651/0278-0372(2001)021[0905:CSCARS]2.0.CO;2

[35] Yen PT, Bart AN. Salinity effects on reproduction of giant freshwater prawn *Macrobrachium rosenbergii* (de Man). Aquaculture. 2008; 280(1–4): 124–128. doi: 10.1016/j.aquaculture.2008.04.035

[36] Zhang S, Dong SL, Wang F. The effects of salinity and food on carbon budget of *Penaeus chinensis*. Journal of Fisheries of China. 1999; 23(2): 144–149 (in Chinese with English abstract).

[37] Wang Q, Zhao YL, Ma Q, Chen LQ. Seasonal changes of biochemical components in reproductive system of male Chinese mitten-Handed crab (*Eriocheir sinensis*). Oceanologia Et Limnologia Sinica. 2004; 35(4): 351–357 (in Chinese with English abstract).

[38] Wei W, Wei H, Liu Q. Effect of estradiol in hemolymph and gonad on precociousness of *Eriocheir sinensis*. Journal of Fisheries of China. 2005; 29(6): 862–865 (in Chinese with English abstract).

[39] Bindhuja MD, Gopal C, Meenakshi M, Revathi K. Effect of salinity on ovarian development of Indian white shrimp *Fenneropenaeus inddicus* (H. Milne edwards, 1837). Journal of Aquaculture in the Tropics. 2013; 28(1–4): 11–19.

[40] Gelin A, Crivelli AJ, Rosecchi E, Kerambrum P. Can salinity changes affect reproductive success in the brown shrimp *Crangon crangon*? Journal of Crustacean Biology. 2001; 21(4): 905–911. doi: 10.1651/0278-0372(2001)021[0905:CSCARS]2.0.CO;2

[41] Bas C, Spivak E. Effect of salinity on embryos of two southwestern Atlantic estuarine grapsid crab species cultured in vitro. Journal of Crustacean Biology. 2000; 20(4): 647–656. doi: 10.1651/0278-0372(2000)020[0647:EOSOEO]2.0.CO;2

[42] Romano N, Zeng C. Osmoregulation in decapod crustaceans: implications to aquaculture productivity, methods for potential improvement and interactions with elevated ammonia exposure. Aquaculture. 2012; 334–337: 12–23. doi: 10.1016/j.aquaculture.2011.12.035

[43] Mo J, Devos P, Trausch G. Active absorption of Cl^-^ and Na^+^ in posterior gills of Chinese mitten crab *Eriocheir sinensis*: Modulation by dopamine and cAMP. Journal of Crustacean Biology. 2003; 23(3): 505–512. doi: 10.1651/C-2295

[44] Feng GP, Lu J, Zhuang P, Wang RF. Effects of salinity on osmo-ionic regulation and enzyme activities in mature female *Eriocheir sinensis*. Marine Fisheries. 2013; 35(4): 468–473 (in Chinese with English abstract). doi: 10.13233/j.cnki.mar.fish.2013.04.015

[45] Torres G, Charmantier-Daures M, Chifflet S, Anger K. Effects of long-term exposure to different salinities on the location and activity of Na^+^/K^+^-ATPase in the gills of juvenile mitten crab, *Eriocheir sinensis*. Comparative Biochemistry and Physiology, Part A: Molecular and Integrative Physiology. 2007; 147(2): 460–465. doi: 10.1016/j.cbpa.2007.01.020 17321769 10.1016/j.cbpa.2007.01.020

[46] Genovese G, Luchetti CG, Luquet CM. Na^+^/K^+^-ATPase activity and gill ultrastructure in the hyper-hypo-regulating crab *Chasmagnathus granulatus* acclimated to dilute, normal and concentrated seawater. Marine Biology. 2004; 144(1): 111–118. doi: 10.1007/s00227-003-1169-6

[47] Lucu Č, Pavičić J, Ivanković D, Pavičić-Hamer D, Najdek M. Changes in Na^+^/K^+^-ATPase activity, unsaturated fatty acids and metallothioneins in gills of the shore crab *Carcinus aestuarii* after dilute seawater acclimation. Comparative Biochemistry and Physiology, Part A: Molecular and Integrative Physiology. 2008; 149(4): 362–372. doi: 10.1016/j.cbpa.2008.01.026 18325806 10.1016/j.cbpa.2008.01.026

[48] Chapelle S, Zwingelstein G. Phospholipid composition and metabolism of crustacean gills as related to changes in environmental salinities: Relationship between Na^+^-ATPase activity and phospholipids. Comparative Biochemistry and Physiology, Part B: Comparative Biochemistry. 1984; 78(2): 363–372. doi: 10.1016/0305-0491(84)90044-0 6088168 10.1016/0305-0491(84)90044-0

[49] Palacios E, Bonilla A, Luna D, Racotta IS. Survival, Na^+^/K^+^-ATPase and lipid responses to salinity challenge in fed and starved white pacific shrimp (*Litopenaeus vannamei*) postlarvae. Aquaculture. 2004; 234(1–4): 497–511. doi: 10.1016/j.aquaculture.2003.12.001

[50] Qi L, Gu XL, Jiang KJ. Effect of salinity on the survival, growth and Na ^+^ /K ^+^ -ATPase activity of early juvenile mud crabs, *Scylla paramamosain*. Marine Science. 2013; 37(2): 56–60 (in Chinese with English abstract).

[51] Romano N, Zeng C. Survival, osmoregulation and ammonia-N excretion of blue swimmer crab, *Portunus pelagicus*, juveniles exposed to different ammonia-N and salinity combinations. Comparative Biochemistry and Physiology, Part C: Toxicology and Pharmacology. 2010; 151(2): 222–228. doi: 10.1016/j.cbpc.2009.10.011 19892035 10.1016/j.cbpc.2009.10.011

[52] Winkler A. Effects of inorganic sea water constituents on branchial Na- K ATPase activity in the shore crab *Carcinus maenas*. Marine Biology. 1986; 92(4): 537–544. doi: 10.1007/BF00392513

[53] Vincent-Marique C, Gilles R. Modification of the amino acid pool in blood and muscle of *Eriocheir sinensis* during osmotic stress. Comparative Biochemistry and Physiology. 1970; 35(2): 479–485. doi: 10.1016/0010-406X(70)90611-0

[54] Al-Azhary DB, Tawfek NS, Meligi NM, Elliottet M. Physiological responses to hyper saline waters in *Necora puber* (Velvet Crab). Pakistan Journal of Physiology. 2008; 4(2): 1–6.

[55] Lorenzon S, Giulianini PG, Ferrero EA. Lipopolysaccharide induced hyperglycemia is mediated by CHH release in crustaceans. General and Comparative Endocrinology. 1997; 108(3): 395–405. doi: 10.1006/gcen.1997.6986 9405116 10.1006/gcen.1997.6986

[56] Wen XB, Chen LQ, Ai CX. Studies on standard metabolism of the parent crab *Eriocheir sinensis*. Journal of East China Normal University (Natural Science). 2002; (3): 105–109 (in Chinese with English abstract).

[57] Luvizotto-Santos R, Lee JT, Branco ZP, Bianchini A, Nery LEM. Lipids as energy source during salinity acclimation in the euryhaline crab *Chasmagnathus granulata* Dana, 1851(Crustacea- Grapsidae). Journal of Experimental Zoology, Part A: Comparative Experimental Biology. 2003; 295(2): 200–205. doi: 10.1002/jez.a.10219 12541304 10.1002/jez.a.10219

[58] Rocatta IS, Palacios E. Hemolymph metabolic variables in response to experimental manipulation stress and serotonin injection in *Penaeus vannamei*. Journal of the World Aquaculture Society. 2000; 29(3): 351–356. doi: 10.1111/j.1749-7345.1998.tb00658.x

[59] Cheng YX, Wang W, Wu JM, Huang XQ. Lipid requirement of decapod crustacean larvae and the relationship between lipid and larval development. Journal of Fishery Science of China. 2000; 7(4): 104–107 (in Chinese with English abstract).

[60] Chen JC, Lin CY. Response of oxygen consumption, ammonia-N excretion and urea-N excretion of *Penaeus chinensis* exposed to ambient at different salinity and pH levels. Aquaculture. 1995; 136(3–4): 243–255. doi: 10.1016/0044-8486(95)01060-2

[61] Chang EWY, Ai ML, Wong WP, Chew SF, Wilson JM, Ip YK. Changes in tissue free amino acid contents, branchial Na^+^/K^+^-ATPase activity and bimodal breathing pattern in the fresh water climbing perch, *Anabas testudineus* (Bloch), during seawater acclimation. Journal of Experimental Zoology, Part A: Ecological Genetics and Physiology. 2007; 307(12): 708–723. doi: 10.1002/jez.a.424 17963240 10.1002/jez.a.424

[62] Lee W, Chen JC. Hemolymph ammonia, urea and uric acid levels and nitrogenous excretion of *Marsupenaeus japonicas* at different salinity levels. Journal of Experimental Marine Biology and Ecology. 2003; 288: 39–49. doi: 10.1016/S0022-0981(02)00597-X

[63] Regnault M. Effect of air exposure on nitrogen metabolism in the crab *Cancer pagurus*. Journal of Experimental Zoology. 1992; 264(4): 372–380. doi: 10.1002/jez.1402640403 1460435 10.1002/jez.1402640403

[64] Weihrauch D, Morris S, Towle DW. Ammonia excretion in aquatic and terrestrial crabs. Journal of Experimental Biology. 2005; 207(26): 4491–4504. doi: 10.1242/jeb.01308 15579545 10.1242/jeb.01308

[65] Li EC, Chen LQ, Zeng C, Yu N, Xiong ZQ, Chen XF, et al Comparison of digestive and antioxidant enzymes activities, hemolymph oxyhemocyanin contents and hepatopancreas histology of white shrimp, *Litopenaeus vannamei*, at various salinities. Aquaculture. 2008; 274(1): 80–86. doi: 10.1016/j.aquaculture.2007.11.001

[66] Li YQ, Li YS, Zhao FZ. Effect of salinity changes on osmotic-, metabolic-, and immune- related enzyme activities in *Exopalaemon carinicauda*. Acta Ecologica Sinica. 2015; 35(2): 7229–7235(in Chinese with English abstract).

[67] Ross SW, Dalton DA, Kranmer S, Christensen BL. Physiological (antioxidant) responses of estuarine fishes to variability in dissolved oxygen. Comparative Biochemistry and Physiology, Part C: Toxicology and Pharmacology. 2001; 130(3): 289–303. doi: 10.1016/S1532-0456(01)00243-5 11701386 10.1016/s1532-0456(01)00243-5

[68] Limdi JK, Hyde GM. Evaluation of abnormal liver function tests. Postgraduate Medical Journal 2003; 79(932): 307–312. doi: 10.1136/pmj.79.932.307 12840117 10.1136/pmj.79.932.307 PMC1742736

[69] Whitfield JB. Gamma glutamyl transferase. Critical Reviews in Clinical Laboratory Sciences. 2001; 38(4): 263–355. doi: 10.1080/20014091084227 11563810 10.1080/20014091084227

[70] Pinoni SA, Goldemberg AL, Mananes AAL. Alkaline phosphatase activities in muscle of the euryhaline crab *Chasmagnathus granulatus*: response to environmental salinity. Journal of Experimental Marine Biology and Ecology. 2005; 326(2): 221–226. doi: 10.1016/j.jembe.2005.06.004

