# Supplementary figures and images for: Effects of salinity on gonadal development, osmoregulation and metabolism of adult male Chinese mitten crab, Eriocheir sinensis
Source: PLoS One. 2017 Jun 19;12(6):e0179036. doi: 10.1371/journal.pone.0179036 (PMC5476241; doi:10.1371/journal.pone.0179036)

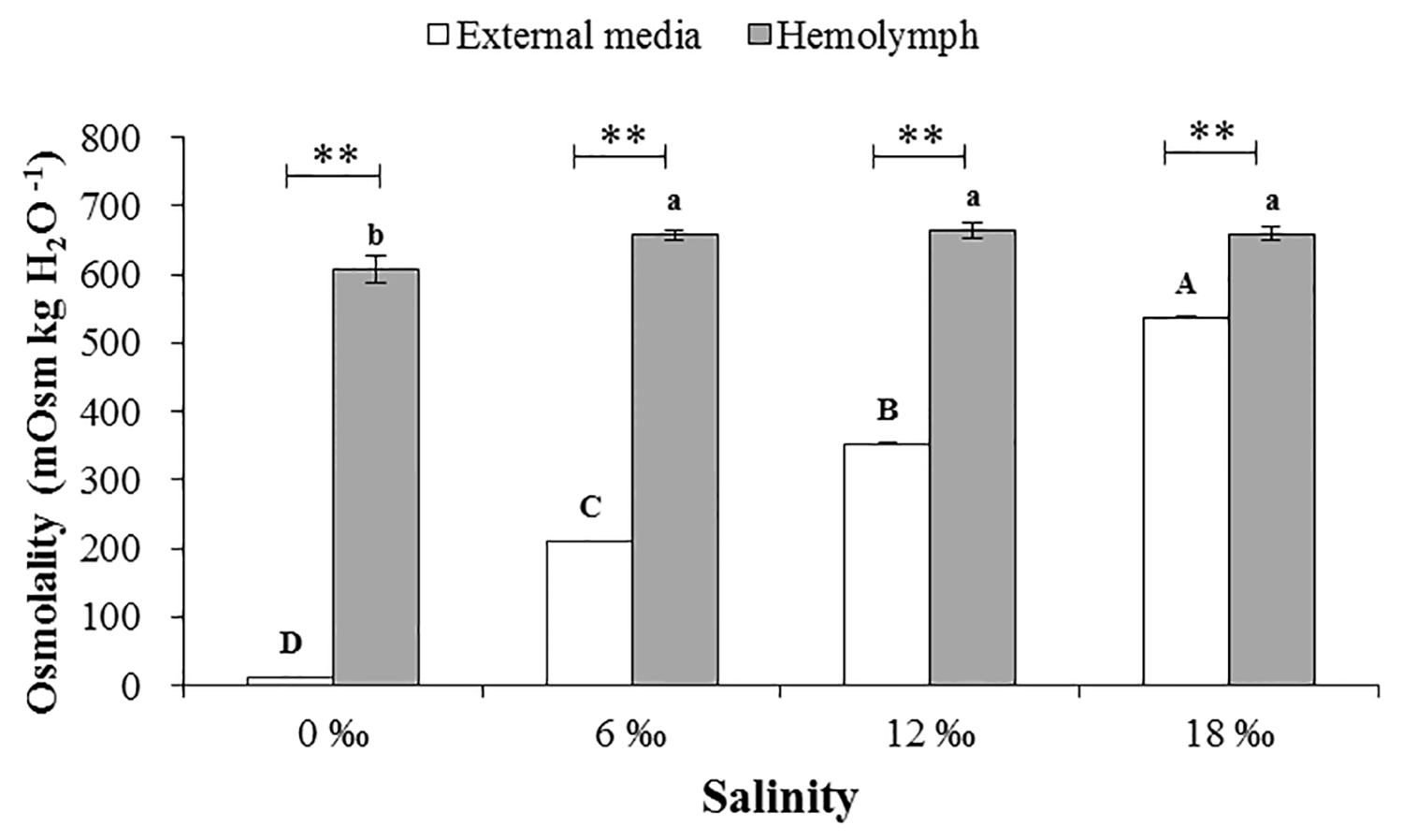

Supplement: S1 Fig — Different lowercase letters “a, b” on the tops of the bars indicated significant differences (P < 0.05) in hemolymph osmolality among different salinity treatments, while the different capital letters “A, B, C, D” indicated significant differences (P < 0.05) in external osmolality among all different salinity treatments. The superscript ‘**’ on the top of the bars indicated vary significant differences (P < 0.01) on the osmolality between the hemolymph of male crabs and their respectively external media. (TIF) [file pone.0179036.s001.tif]
